# Characterizing worker compensation claims in long-term care and examining the association between facility characteristics and severe injury: a repeated cross-sectional study from Alberta, Canada

**DOI:** 10.1186/s12960-023-00850-4

**Published:** 2023-08-16

**Authors:** Stephanie A. Chamberlain, Fangfang Fu, Oludotun Akinlawon, Carole A. Estabrooks, Andrea Gruneir

**Affiliations:** 1 Faculty of Nursing, College of Health Sciences, University of Alberta, Edmonton, AB Canada; 2 Department of Family Medicine, Faculty of Medicine and Dentistry, College of Health Sciences, University of Alberta, Edmonton, AB Canada

**Keywords:** Workforce characteristics, Long-term care, Workplace safety

## Abstract

**Background:**

Despite the physical demands and risks inherent to working in long-term care (LTC), little is known about workplace injuries and worker compensation claims in this setting. The purpose of this study was to characterize workplace injuries in LTC and to estimate the association between worker and organizational factors on severe injury.

**Methods:**

We used a repeated cross-sectional design to examine worker compensation claims between September 1, 2014 and September 30, 2018 from 25 LTC homes. Worker compensation claim data came from The Workers Compensation Board of Alberta. LTC facility data came from the Translating Research in Elder Care program. We used descriptive statistics to characterize the sample and multivariable logistic regression to estimate the association between staff, organizational, and resident characteristics and severe injury, measured as 31+ days of disability.

**Results:**

We examined 3337 compensation claims from 25 LTC facilities. Less than 10% of claims (5.1%, n = 170) resulted in severe injury and most claims did not result in any days of disability (70.9%, n = 2367). Most of the sample were women and over 40 years of age. Care aides were the largest occupational group (62.1%, n = 2072). The highest proportion of claims were made from staff working in voluntary not for profit facilities (41.9%, n = 1398) followed by public not for profit (32.9%, n = 1098), and private for profit (n = 25.2%, n = 841). Most claims identified the nature of injury as traumatic injuries to muscles, tendons, ligaments, or joints. In the multivariable logistic regression, higher staff age (50–59, aOR: 2.26, 95% CI 1.06–4.83; 60+, aOR: 2.70, 95% CI 1.20–6.08) was associated with more severe injury, controlling for resident acuity and other organizational staffing factors.

**Conclusions:**

Most claims were made by care aides and were due to musculoskeletal injuries. In LTC, few worker compensation claims were due to severe injury. More research is needed to delve into the specific features of the LTC setting that are related to worker injury.

**Supplementary Information:**

The online version contains supplementary material available at 10.1186/s12960-023-00850-4.

## Background

Risk of workplace injury is among the highest in healthcare settings [1, 2]. Long-term care (LTC) homes (i.e., nursing homes) are one of the most dangerous workplaces and have one of the highest rates of occupational injury [3]. High rates of injury in LTC are partly (but not entirely) owed to the characteristics of the care recipients in the setting. Residents cared for in LTC homes are typically older adults who require assistance with nearly all activities of daily living, most have moderate to severe cognitive impairment, functional limitations, and complex co-morbidities [4–7]. Caring for residents includes tasks such as assisting with transfers, repositioning, and providing personal care (toileting, dressing, feeding, and bathing) to residents who may be unresponsive or uncooperative [8–10]. Unsurprisingly, care aides, who provide the bulk of such care, have the highest risk of occupational injury [11, 12]. Occupational injury in LTC is a risk to the sector as it struggles to cope with increased demands for LTC services and an aging workforce. Fear of being seriously injured on the job increases the likelihood of employee turnover and intention to leave, both of which are critical issues in the LTC sector that already deals with persistent worker shortages [13]. Little is known about the characteristics of LTC staff who are seriously injured and this study aims to describe these characteristics and identify factors that might contribute to serious injury in the LTC workforce.

The quality of the work environment in LTC homes has been widely studied because of its variability and its effects on resident care and organizational performance [14]. A poor work environment has negative effects on staff (e.g., burnout, satisfaction, mental and physical health) [15–17] and residents (e.g., pressure ulcers, hospitalizations, missed and rushed care) [18, 19]. Comparatively less is known about how the work environment and other organizational factors might also influence worker injury and resulting worker compensation claims [20, 21]. Research has found that a poor workplace safety climate increases the likelihood of employee dissatisfaction and turnover intention [13]. Unlike resident characteristics, many features of the work environment are modifiable, yet their relationship to worker injury is largely unknown. The aim of this study was to examine the staff characteristics and organizational factors related to injury claims in LTC homes. In this study, we linked worker compensation claim data and LTC home data to describe worker and injury characteristics and to estimate the association between LTC home features and organizational context on the severity of worker injury in Alberta, Canada.

## Methods

### Data sources

This retrospective repeated cross-sectional study used worker compensation claims in Alberta, Canada. Worker compensation claim data came from The Workers Compensation Board (WCB) of Alberta which delivers workplace insurance to provincial employers and provides financial benefits and reimburses health care costs for individuals experiencing a work-related injury [22]. The WCB collects information on claimants (age, sex, occupation), the nature of the incident that triggered the claim (accident type, location), injury type, and subsequent days of disability. Data on LTC facility characteristics came from the Translating Research in Elder Care (TREC) research program, which aims to improve resident care and staff work life [23, 24]. TREC collects resident, staff, and facility data in 94 LTC homes in Alberta, British Columbia, and Manitoba. LTC homes in the TREC program are randomly selected to be representative of LTC homes in urban areas and are proportionally stratified by region, ownership model (public not for profit, private for profit, voluntary not for profit) and bed size (small: 35–79, medium: 80–120, large: > 120). Of the 94 homes in the TREC cohort, 25 are from Alberta and therefore linkable to WCB data.

The TREC program administers a suite of survey instruments, collectively known as the TREC Survey, to staff. The TREC Survey collects information on staff demographics, physical and mental health, and quality of work life. The TREC Survey also includes the Alberta Context Tool (ACT) a survey-based instrument developed and validated to assess a facility’s organizational context and readiness for best practice uptake [23–25]. TREC collects resident data from each participating facility. Resident data are collected using the Resident Assessment Instrument-Minimum Data Set (RAI-MDS 2.0) [26]. The RAI-MDS is completed in LTC homes and is a comprehensive longitudinal assessment tool that collects information on clinical and functional status of residents. It is completed upon admission, quarterly, following a significant change in resident health, and at discharge. Assessments include details such as resident demographics, physical functioning, cognition, and disease diagnoses. The benefit of WCB linkage to data from the TREC program was that we could ascertain resident acuity, facility structure (ownership, bed size, staffing numbers), and organizational context (work environment) data, otherwise not available in the WCB data. Each claim was linked to the specific LTC facility where the injury was reported, using unique TREC facility codes that could be applied back to the WCB data.

### Study sample

**Fig. 1:**
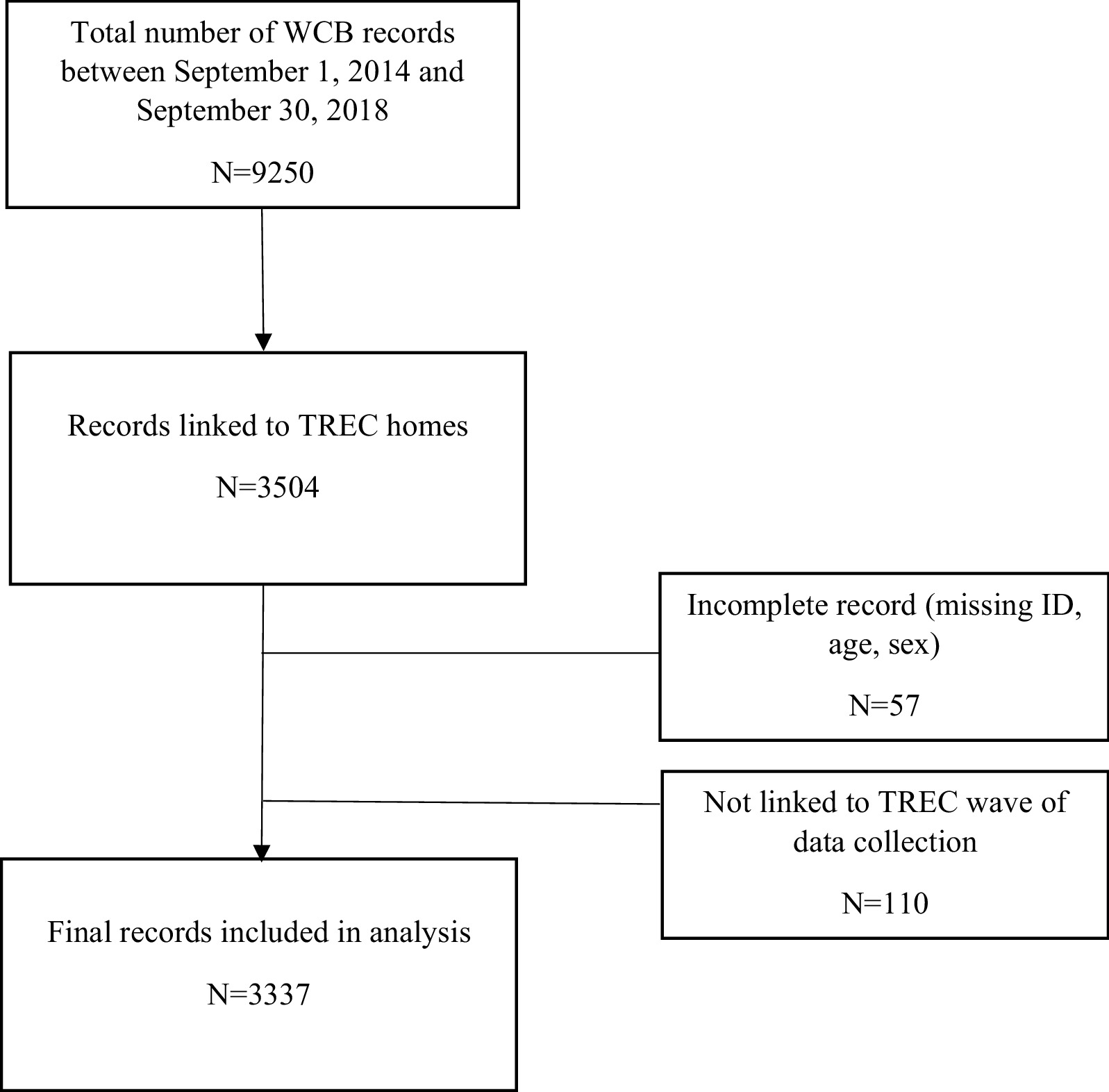
Sample flow chart

We received all Alberta WCB claims from September 1, 2014 to September 30, 2018 (n = 9250). We excluded records where the claim was not within 1 year of a wave of TREC data collection (waves occurred on September 8, 2014 to May 15, 2015 and May 1, 2017 to December 1, 2017), the claimant did not have a valid personal identifier, or was missing age, sex, or total days of disability (n = 167). This resulted in a final sample of 3337 WCB claims between September 1, 2014 and September 30, 2018 from the 25 Alberta LTC homes in the TREC cohort (Fig. 1).

### Outcome variable: severe injury

We examined total days of disability in three exclusive categories: no days, < 31 days, 31+ days. In the regression analysis, we collapsed no days and < 31 days as the reference category and examined the odds of 31+ days of disability. We defined severe injury as having 31 or greater days of disability. We selected 31+ days of disability because this is the time frame when WCB claims in Alberta are transitioned to long-term disability and require specialist case management. Other research examining Alberta WCB data has used similar time loss categories to describe worker injury [27]. Duration of disability (e.g., > 31 days off) has been described as a proxy for injury severity whereby more days of disability reflect a more serious injury [28]. Severe injury is important to examine because it is a driver of disability claims and severe injury increases the likelihood of leaving the workforce. Fears of serious injury are associated with turnover and turnover intention. Fewer days of disability can indicate that the injury was minor and required no workplace modifications.

### Independent variables: organizational characteristics

We examined organizational characteristics including LTC facility ownership (public not for profit, private for profit, voluntary not for profit), facility bed size (small: < 80 beds, medium: 80–120 beds, large: > 120 beds), and the total care aide workforce in the facility (31–79, 80–120, 121+). We assessed potentially modifiable elements of the organizational context (work environment) using the ACT [23, 24]. The ACT measures ten concepts of organizational context (leadership, culture, evaluation, social capital, informal interactions, formal interactions, resources, organizational slack-staff, organizational slack-time, organizational slack-space). The validity of the ACT has been assessed in the LTC setting using confirmatory factor analysis, analysis of variance, and bivariate associations between each concept and research use. Individual care aide scores can be validly aggregated at the facility level [29]. We categorized the cumulative ACT score into quartiles (quartile 1: lowest context, quartile 2: low context, quartile 3: high context, and quartile 4: highest context) [30]. The higher the organizational context, the more favourable the work environment.

### Other covariates

Claimant demographics were obtained from the WCB data and included age (18–29, 30–39, 40–49, 50–59, 60+), sex (female, male), and occupation (unregulated care aide, regulated health professional, food service, housekeeping and maintenance, other support staff, not yet determined). We also reported on the claimant’s primary source of injury, nature of injury, part of body, and number of claims during observation period. It should be noted that claims data could not be linked to individual TREC survey respondents.

Characteristics about resident physical functioning and cognition were obtained from the RAI-MDS. We used the Activities of Daily Living-Hierarchy (ADL-H) Scale to assess resident physical functioning [31] and the Cognitive Performance Scale (CPS) to assess residents’ cognitive status [32]. Higher scores in each scale indicated worsening performance.

### Analysis

We selected variables based on availability in the WCB database and research on LTC organizational context and staff outcomes [15, 18, 19]. We calculated descriptive statistics (frequencies, percent) for all variables. The multivariable logistic regression examined the odds of severe injury (reference: no/less than 31 days of disability) and included all the available facility characteristics and organizational context details and adjusted for claimant and resident characteristics. To adjust for resident acuity, for each facility we calculated the percent of residents with moderate-severe cognitive impairment (CPS score of 3+) and the percent of residents with the highest level of functional impairment (ADL-H score of 5+).

Our primary logistic regression analysis included all claims from September 1, 2014 to September 30, 2018, regardless of the claimant occupation. As a sensitivity analysis, we examined only claimants identified as care staff, which included the following care staff position codes: nurse aides, orderlies, and patient service associates, registered nurses, registered psychiatric nurses, and assisting occupations in support of health services, licensed practical nurses, managers in health care, and nursing coordinators and supervisors (see Additional file 1). The reason for the sensitivity analysis was that while we anticipate that organizational factors could influence injury experience regardless of role, we wanted to examine the specific association between organizational factors and severe injury for care staff in regular and direct contact with residents. All analysis was conducted in SAS 9.4 (SAS Institute, Cary, NC).

## Results

Our sample included 3337 claims from 25 LTC homes. We found that 5.1% of claims (n = 170) resulted in severe injury (31+ days of disability), and most claims did not result in any days of disability (70.9%, n = 2367) (Table 1). Most claims were attributed to women (90.7%, n = 3028) and those 40+ years of age (73%, n = 2436), with the highest proportion between 50 and 59 years of age. Care aides were the largest occupational group (62.1%, n = 2072). They accounted for the highest percentage of claims resulting in severe injury (67.7%, n = 115) followed by regulated health professionals (13.5%, n = 23), and housekeeping and maintenance (8.8%, n = 15).

**Table 1:** Worker characteristics (n = 3337) by total days of disability from 25 long-term care homes in Alberta, Canada between September 1, 2014 and September 30, 2018

	Total days of disability			
	None N (%)	< 31 days N (%)	31+ days N (%)	Total N (%)
	N = 2367 (70.9%)	N = 800 (24.0%)	N = 170 (5.1%)	N = 3337 (100.0%)
Sex				
Female	2163 (91.4)	710 (88.8)	155 (91.2)	3028 (90.7)
Male	204 (8.6)	90 (11.3)	15 (8.8)	309 (9.3)
Age, mean (SD)	46.5 (11.3)	46.3 (11.2)	48.9 (10.2)	46.6 (11.2)
Age				
18–29	203 (8.6)	75 (9.4)	7 (4.1)	285 (8.5)
30–39	443 (18.7)	146 (18.3)	27 (15.9)	616 (18.5)
40–49	693 (29.3)	233 (29.1)	48 (28.2)	974 (29.2)
50–59	741 (31.3)	250 (31.3)	60 (35.3)	1051 (31.5)
60+	287 (12.1)	96 (12.0)	28 (16.5)	411 (12.3)
Occupation				
Unregulated care aide	1420 (60.0)	537 (67.1)	115 (67.7)	2072 (62.1)
Regulated health professional	359 (15.2)	113 (14.1)	23 (13.5)	495 (14.8)
Food service	156 (6.6)	47 (5.9)	6 (3.5)	209 (6.3)
Housekeeping and maintenance	177 (7.5)	54 (6.8)	15 (8.8)	246 (7.4)
Other support staff	184 (7.8)	45 (5.6)	10 (5.9)	239 (7.2)
Not yet determined	71 (3)	0–5	0–5	76 (2.3)
Primary source of injury				
Person	1219 (51.5)	428 (53.5)	116 (68.2)	1763 (52.8)
Structures and surfaces	445 (18.8)	147 (18.4)	33 (19.4)	625 (18.7)
Tools, instruments, and equipment	405 (17.1)	98 (12.3)	15 (8.8)	518 (15.5)
Plant, animal, bacteria, chemicals	124 (5.2)	106 (13.3)	0–5	230 (6.9)
Not yet determined, unknown	174 (7.4)	21 (2.6)	6 (3.5)	201 (6.0)
Type of accident				
Bodily reaction and exertion	1021 (43.1)	411 (51.4)	116 (68.2)	1548 (46.4)
Contacts with objects and equipment	427 (18.0)	166 (20.8)	12 (7.1)	605 (18.1)
Assaults and violent acts	392 (16.6)	90 (11.3)	17 (10.0)	499 (15)
Falls	275 (11.6)	103 (12.9)	25 (14.7)	403 (12.1)
Exposure to harmful substances or environments	139 (5.9)	23 (2.9)	0–5	162 (4.9)
Not yet determined, unknown	113 (4.8)	7 (0.9)	0–5	120 (3.6)
Nature of injury				
Traumatic injuries to muscles, tendons, ligaments, joints	1265 (53.4)	500 (62.5)	128 (75.3)	1893 (56.7)
Wounds, bruises, burns	645 (27.3)	112 (14.0)	10 (5.9)	767 (23)
Other traumatic injuries and disorders	299 (12.6)	102 (12.8)	26 (15.3)	427 (12.8)
Other diseases	70 (3.0)	72 (9.0)	5 (2.9)	147 (4.4)
Symptoms, signs, and ill-defined conditions	49 (2.1)	8 (1.0)	0–5	58 (1.7)
Unknown, not yet determined	29 (1.2)	0–5	0–5	29 (0.9)
Infectious and parasitic diseases	10 (0.4)	6 (0.8)	0–5	16 (0.5)
Injured part of body				
Back, including spine, spinal cord	407 (17.2)	227 (28.4)	46 (27.1)	680 (20.4)
Hands and fingers	380 (16.1)	50 (6.3)	7 (4.1)	437 (13.1)
Shoulder including clavicle, scapula, and trapezius muscle	288 (12.2)	109 (13.6)	26 (15.3)	423 (12.7)
Multiple body parts	205 (8.7)	76 (9.5)	21 (12.4)	302 (9.1)
Legs and ankles	188 (7.9)	62 (7.8)	23 (13.5)	273 (8.2)
Arms	207 (8.8)	39 (4.9)	13 (7.7)	259 (7.8)
Chest, including ribs, internal organs, neck, abdomen, trunk, upper extremities	180 (7.6)	63 (7.9)	12 (7.1)	255 (7.6)
Head, face, ears, cranial region including skull	194 (8.2)	44 (5.5)	7 (4.1)	245 (7.3)
Wrists	157 (6.6)	29 (3.6)	11 (6.5)	197 (5.9)
Body systems	55 (2.3)	82 (10.3)	0–5	139 (4.2)
Foot (feet), toe (toes)	58 (2.5)	19 (2.4)	0–5	79 (2.4)
Unknown, not yet determined	48 (2.0)	0–5	0–5	48 (1.4)
Number of claims per claimant				
1 claim	1199 (50.7)	403 (50.4)	81 (47.7)	1683 (50.4)
2+ claims	1168 (49.4)	397 (49.6)	89 (52.4)	1654 (49.6)

The primary source of injury for all claims, regardless of injury severity, was a person (n = 1763, 52.8%), and this was more common among claims with severe injury (31+ days of disability) than those with no days of disability (68.2% versus 51.5%). Regardless of total days of disability, nearly half of claims reported that the type of accident responsible for the injury was bodily reaction and exertion (46.4%, n = 1548). When we examined the type of accident by total days of disability, different accident patterns emerge. Claims with 31+ days of total disability had the highest percentage attributed to falls (14.7%, n = 25), and claims with no days of disability had the highest percentage attributed to assaults and violent acts (16.6%, n = 392). Most claims identified the nature of injury as traumatic injuries to muscles, tendons, ligaments, or joints. The proportion of claims that reported these traumatic injuries increased as total days of disability increased. Claims with no days of disability showed the greatest prevalence of wounds and burns (27.3%, n = 645). Over 20% (20.4%, n = 680) of all claims reported a back injury, the highest proportion in those with < 31 days of disability. Claims for injuries of multiple body parts had the most total days of disability.

A total of 25 LTC facilities were represented in our sample. Most of the facilities in the sample were profit-for-profit (n = 12) and had more than 120 beds (n = 17), characteristics which are consistent with the LTC sector in Alberta. Resident acuity (physical functioning, cognitive status) did not differ significantly across the facilities in our sample or by injury severity. When examining organizational characteristics and worker claims, the highest proportion of claims were made from staff working in voluntary not for profit facilities (41.9%, n = 1398) followed by public not for profit (32.9%, n = 1098), and private for profit (n = 25.2%, n = 841). This descriptive pattern was observed claims of 1–30 and 31+ days, as well. Most claims came from larger facilities with more than 120 beds but this did not differ based on days of disability. LTC homes with a more favourable (highest quartile) organizational context had the highest proportion of all claims (42.9%, n = 1431). We did not observe differences in the number of claims by the total care aide workforce in the facility (Table 2).

**Table 2:** Organizational characteristics by total days of disability from 25 long-term care homes in Alberta, Canada between September 1, 2014 and September 30, 2018

	Total days of disability			
	None N (%)	< 31 days N (%)	31+ days N (%)	Total N (%)
	N = 2367 (70.9%)	N = 800 (24.0%)	N = 170 (5.1%)	N = 3337 (100.0%)
Ownership				
Public not for profit (n = 7)	783 (33.1)	265 (33.1)	50 (29.4)	1098 (32.9)
Private for profit (n = 12)	641 (27.1)	167 (20.9)	33 (19.4)	841 (25.2)
Voluntary not for profit (n = 6)	943 (39.8)	368 (46.0)	87 (51.2)	1398 (41.9)
Bed size				
Small (< 80 beds) (n = 2)	52 (2.2)	22 (2.8)	5 (2.9)	79 (2.4)
Medium (80–120 beds) (n = 6)	289 (12.2)	77 (9.6)	17 (10.0)	383 (11.5)
Large (> 120 beds) (n = 17)	2026 (85.6)	701 (87.6)	148 (87.1)	2875 (86.2)
Organizational context				
Quartile 1: lowest context	417 (17.6)	115 (14.4)	37 (21.8)	569 (17.1)
Quartile 2: low context	611 (25.8)	203 (25.4)	45 (26.5)	859 (25.7)
Quartile 3: high context	346 (14.6)	114 (14.3)	18 (10.6)	478 (14.3)
Quartile 4: highest context	993 (42.0)	368 (46.0)	70 (41.2)	1431 (42.9)
Total care aide workforce in facility				
Mean, SD	101.7 (38.9)	103.9 (40.5)	101.6 (38.1)	102.2 (39.3)
31–79	892 (37.7)	277 (34.6)	55 (32.4)	1224 (36.7)
80–120	721 (30.5)	234 (29.3)	60 (35.3)	1015 (30.4)
121 and above	754 (31.9)	289 (36.1)	55 (32.4)	1098 (32.9)
Resident characteristics				
% of resident with moderate-severe cognitive impairment (CPS^a^ 3+), Mean (SD)	73.7 (8.8)	73.7 (7.6)	73.3 (8.4)	73.6 (8.5)
% of residents with highest level of ADL impairment (Adl-H^b^ 5+), mean (SD)	47.8 (15.2)	47.3 (15.6)	45.5 (14.2)	47.6 (15.3)

^a^Cognitive Performance Scale

^b^Activities of Daily Living-Hierarchy Scale

In the multivariable logistic regression, we found that higher staff age was associated with more severe disability (31+ days versus < 30 days) (Table 3). Our adjusted models show that older staff (50–59 and 60+) when compared to younger staff (18–29) had greater odds of more than 31 days of disability (OR = 2.26 and OR = 2.70, respectively), controlling for other resident, organizational, and staffing factors. We found only slight differences based on organizational context and percent of residents with higher ADL impairments, but they were approaching insignificance. We conducted a sensitivity analysis examining only the sample that were care staff (n = 2609) and this did not show any significant associations (Additional file 1).

**Table 3:** Multivariable logistic regression analysis estimating the association between claimant characteristics and organizational factors on severe injury (31+ days of disability) from 3337 worker compensation claims in Alberta between September 1, 2014 and September 30, 2018

Variable	Unadjusted			Adjusted		
	Odds ratio	95% Confidence interval	P-value	Odds ratio	95% Confidence interval	P-value
Most recent wave of data collection (reference: wave 3 = previous wave)	1.14	0.83–1.57	0.4300	1.15	0.82–1.60	0.4254
Bed size (reference = small < 80 beds)						
Large (> 120 beds)	0.74	0.30–1.79	0.5003	0.61	0.23–1.64	0.3287
Medium	0.65	0.24–1.75	0.3901	0.75	0.27–2.11	0.5816
Total care aide workforce (reference = 31–79)						
121 and above	1.12	0.77–1.64	0.5581	1.20	0.77–1.87	0.4310
80–120	1.33	0.92–1.94	0.1311	1.28	0.82–1.98	0.2753
Owner-operator (reference = public not for profit)						
Private for profit	0.86	0.55–1.34	0.5089	0.54	0.28–1.07	0.0779
Voluntary not for profit	1.39	0.97–1.98	0.0727	0.95	0.55–1.66	0.8668
Organizational context (reference = Q1, lowest context score quartile)						
Q2-low context	0.79	0.51–1.24	0.3090	0.70	0.42–1.15	0.1570
Q3-high context	0.57	0.32–1.01	0.0541	0.54	0.29–0.99	0.0480
Q4-highest context	0.74	0.49–1.11	0.1405	0.69	0.41–1.17	0.1688
Claimant age (reference = 18–29)						
30–39	1.73	0.76–3.94	0.1897	1.67	0.74–3.75	0.2167
40–49	1.94	0.89–4.25	0.0957	1.94	0.90–4.19	0.0930
50–59	2.27	1.05–4.90	0.0377	2.26	1.06–4.83	0.0359
60+	2.76	1.22–6.27	0.0153	2.70	1.20–6.08	0.0165
Claimant sex (reference = male)						
Female	1.03	0.60–1.76	0.9197	1.02	0.60–1.74	0.9397
% of resident with moderate-severe cognitive impairment (CPS 3+)^a^	0.62	0.10–3.71	0.5982	0.45	0.03–6.95	0.5662
% residents with highest level of ADL impairment (ADL-H 5+)^b^	0.39	0.14–1.10	0.0758	0.20	0.04–0.94	0.0419

^a^Cognitive Performance Scale

^b^Activities of Daily Living-Hierarchy Scale

## Discussion

We examined descriptive characteristics of WCB claims in 25 Alberta LTC homes. Claimants were typically older and overwhelmingly female. Our descriptive finding that WCB claims were primarily from older women is not at all surprising given that the LTC workforce is primarily female and an older workforce with most over the age of 50 [33]. Most claims came from staff working in voluntary not for profit owned facilities with > 120 beds. We did not find other significant associations in our multivariable model between facility or organizational context characteristics and severity of injury.

Results from our multivariable logistic regression found that compared to younger staff (18–29 years), staff over 50 years of age had higher odds of severe injury. While the workforce overall may be older and have poorer health than the general population [34], it is not clear from these data how exactly claimant age is related to severe injury. For example, risk of severe injury may be due to age-related changes in care aides’ musculoskeletal systems, repetitive strains, or prolonged exposure to injury inducing conditions. Because we were unable to identify individual claims with the TREC facility data, we cannot assess the length of time staff worked in the facility. It is likely that older staff have worked in the position for longer than younger staff, and this prolonged tenure in the role and duration of injury inducing work may contribute to their increased risk of severe injury. Irrespective of the underlying reasons for the association between increased staff age and severe injury, our findings point to the need for workforce planning. The LTC sector must grapple with an aging workforce that is at heightened risk for severe injury, frequently working short staffed, and caring for an increasingly dependent and complex resident population [35]. Workforce planning is needed to recruit younger workers to the sector and to retain and retrain workers who have been working in direct care roles.

The most reported injury in our sample was traumatic injuries to muscles, tendons, ligaments, or joints. Over 20% of claims in our sample were due to a back injury. This is consistent with other work in LTC settings indicating that direct care staff injuries are primarily due to musculoskeletal issues [36]. Cohen-Mansfield, Culpepper, and Carter [37] found that back injuries comprised over 75% of all injuries in LTC. Most back injuries were due to overexertion (66.5%) followed by being struck by an object. Lifting is a frequent activity that results in injury [37]. Nearly half of our claimants reported that ‘type of accident’ responsible for their injury was bodily reaction and exertion. Bodily reactions and overexertion reflect injuries sustained through excessive physical efforts which may include repetitive or awkward movements such as bending, twisting, or reaching [3]. The type of accident and resulting muscular injuries are consistent with the type of physical activities that LTC staff engage in when caring for many residents during their shift including dressing, bathing, lifting, and repositioning. Given the type of activities that care aides engage in, it is not surprising that most claims in our cohort were made by care aides, followed by regulated care providers.

Our descriptive analysis showed, somewhat counterintuitively, that homes with a higher organizational context (more favourable work environment) had the highest frequency of claims. This finding is inconsistent with research that examined variation in staff injury rates in intermediate care facilities and found that facilities with more favourable staffing levels and supportive work environments (e.g., supportive management, worker perception of employer fairness) had lower staff injury rates [38]. Our descriptive results suggest that homes with a more favourable work environment had more claims may be due to organizations fostering a safety climate that encouraged greater reporting of actual and potential injuries.

Our regression results found only minimally significant effects between organizational context and severe disability. This may be due to our inability to measure more specific elements of safety culture and feelings of job security. We found that a greater proportion of all claims and claims for severe injury came from not for profit than for profit facilities, but we found no significant association following adjustment for resident acuity and other staffing variables. This is somewhat unexpected given U.S. research that found that for-profit facilities had higher staff injury rates [39]. These descriptive differences in staff injury and organizational features (e.g., work environment, ownership) could be due to differences in owner-operator models that influence the staff’s perception of job security if they report an injury. We know that reported claims are likely an underestimation of the total burden of staff injury in LTC because WCB data does reflect unapproved claims or when a worker is injured but chooses not to file a claim. Research indicates that nearly 40% of eligible claims are not reported to a compensation board and board records do not capture injuries not covered by insurance [40, 41]. Interviews with injured workers and service providers identified four main areas that play a role in complicating, prolonging, or choosing not to pursue compensation claims. These relate to health care access, limited understanding of the compensation system requirements, confusion about the claim process, and decision-making authorities [42]. Systematic underreporting could also be explained by other factors including time pressures, worker doubts about eligibility (perceptions of ineligibility for benefits), reputation, income loss, and career prospects [43]. Both perceived ineligibility (the belief that injuries are part of the job or not serious enough to warrant attention) and concerns about reputation that could be perceived to lead to job or overtime loss are particularly salient for the LTC workforce and may have contributed to the differences in frequency of claims observed by organization features [43].

Care aides, those at greatest risk of injury, often face precarious employment, with nearly a quarter of care aides working at more than one home [44]. Ensuring that such staff recognize the importance of injury reporting and that any real or perceived repercussions of reporting are minimized should be central to worker safety protocols and would help to better understand the extent to which organizational factors are associated with workplace injury frequency and severity. While our data were collected from 2014 to 2018, they provide a baseline understanding of LTC worker injuries and demonstrate the possibility and continued need for WCB claim data linkage with LTC facility and organizational data. Our findings are perhaps even more relevant now considering the current context of LTC and the COVID-19 pandemic. LTC homes in Canada were the site of the highest proportion of COVID-19 deaths and the pandemic made visible the staffing, resource, and data issues in the sector [45]. We lack robust data collection on LTC staffing, notably for staff events and absences across LTC facilities. This study offers a glimpse into these data gaps and opportunities for future linkage.

## Strengths and limitations

Our study examined WCB data in a stratified representative sample of LTC homes. We were able to link both resident, staff, and facility characteristics with WCB data to capture a more comprehensive overview of claims than is otherwise possible. We only examined claims data from one province and are unable to make direct comparisons to other provinces. While the linkage to the TREC cohort offered a unique opportunity to examine resident, staff, and facility characteristics not captured by the WCB, it resulted in a loss of cases because we did not link cases outside waves of data collection. A notable challenge of examining WCB data in the LTC sector relates to the complicated and patchwork reporting on staffing. There are no national and few provincial registries of care aides, the largest workforce in the sector, making it challenging to know the size of the workforce and how much our data might underestimate worker injuries. Tracking injuries across the care aide is further complicated by the fact that many work at more than one facility and/or work casually through an agency [33].

## Conclusion

We examined staff demographics and organizational factors related to severe injury in LTC homes in Alberta, Canada. LTC homes with a more favorable work environment had the highest proportion of claims compared to homes with a less favorable work environment. Although largely descriptive, our work suggests that a better understanding of organizational features and work environment is needed to understand factors that contribute to staff injury and injury reporting.

### Supplementary Information

**Additional file 1: Table S1.** Multivariable logistic regression of direct care staff (n = 2609).

## Data Availability

The datasets used and analyzed for this study are currently housed in the secure and confidential Health Research Data Repository (HRDR) in the Faculty of Nursing at the University of Alberta (https://www.ualberta.ca/nursing/research/supports-and-services/hrdr), in accordance with the health privacy legislation of relevant health jurisdictions. The data that support the findings of this study are available from the Worker Compensation Board (WCB) of Alberta and the Translating Research in Elder Care (TREC) team, but restrictions apply to the availability of the data, which were used as per a data sharing agreement for the study and therefore not publicly available. Information about the study data are available upon reasonable request through the TREC data manager (joseph.akinlawon@ualberta.ca).

## Abbreviations

TREC: Translating Research in Elder Care
WCB: Workers Compensation Board
LTC: Long-term care
