# Supplementary material for: Characterizing worker compensation claims in long-term care and examining the association between facility characteristics and severe injury: a repeated cross-sectional study from Alberta, Canada
Source: Hum Resour Health. 2023 Aug 16;21:63. doi: 10.1186/s12960-023-00850-4 (PMC10433635; doi:10.1186/s12960-023-00850-4)
Supplement: Supplementary file 1 — Additional file 1: Table S1. Multivariable logistic regression of direct care staff (n = 2609). [file 12960_2023_850_MOESM1_ESM.docx]

**Additional file 1**

Table S1. Multivariable logistic regression of direct care staff (n=2609)

| **Variable** | **Unadjusted** | | | **Adjusted** | | |
| --- | --- | --- | --- | --- | --- | --- |
|  | **Odds Ratio** | **Confidence Interval** | **P-value** | **Odds Ratio** | **Confidence Interval** | **P-value** |
| **Most recent wave of data collection** (reference: Wave 3=previous wave) | 1.15 | 0.80 - 1.64 | 0.4583 | 1.15 | 0.79 - 1.68 | 0.4682 |
|  |  |  |  |  |  |  |
| **Bed Size** (reference=small <80 beds) |  | a |  |  |  |  |
| Large (>120 beds) | 0.64 | 0.26 - 1.57 | 0.3261 | 0.42 | 0.16 - 1.11 | 0.0791 |
| Medium (80-120 beds) | 0.58 | 0.21 - 1.60 | 0.2925 | 0.51 | 0.18 - 1.44 | 0.2067 |
|  |  |  |  |  |  |  |
| **Total care aide workforce** (reference=31-79) |  |  |  |  |  |  |
| 121 and above | 1.03 | 0.68 - 1.55 | 0.8963 | 1.24 | 0.76 - 2.02 | 0.3972 |
| 80-120 | 1.27 | 0.84 - 1.91 | 0.2531 | 1.42 | 0.89 - 2.26 | 0.1389 |
|  |  |  |  |  |  |  |
| **Owner-operator** (reference=public not for profit) |  |  |  |  |  |  |
| Private for profit | 0.71 | 0.42 - 1.19 | 0.1908 | 0.69 | 0.40 - 1.18 | 0.1693 |
| Voluntary not for profit | 1.41 | 0.96 - 2.07 | 0.0801 | 1.28 | 0.79 - 2.07 | 0.3229 |
|  |  |  |  |  |  |  |
| **Organizational context** (reference=Q1, lowest context score quartile) |  |  |  |  |  |  |
| Quartile 2: Low Context | 0.79 | 0.49 -1.28 | 0.3435 | 0.74 | 0.43 - 1.25 | 0.2536 |
| Quartile 3: High Context | 0.54 | 0.29 -0.99 | 0.0474 | 0.56 | 0.29 - 1.05 | 0.0714 |
| Quartile 4: Highest Context | 0.64 | 0.41 -1.00 | 0.0510 | 0.60 | 0.34 - 1.04 | 0.0677 |
|  |  |  |  |  |  |  |
| **Claimant Age** (reference=18-29) |  |  |  |  |  |  |
| 30-39 | 1.76 | 0.73 - 4.24 | 0.2105 | 1.72 | 0.72 - 4.10 | 0.2215 |
| 40-49 | 2.08 | 0.90 - 4.80 | 0.0864 | 2.18 | 0.96 - 4.98 | 0.0642 |
| 50-59 | 2.21 | 0.96 - 5.10 | 0.0618 | 2.24 | 0.99 - 5.11 | 0.0543 |
| 60+ | 2.37 | 0.96 - 5.87 | 0.0623 | 2.36 | 0.96 - 5.80 | 0.0601 |
|  |  |  |  |  |  |  |
| **Claimant Sex** (reference=male) |  |  |  |  |  |  |
| Female | 1.82 | 0.76 - 4.34 | 0.1767 | 1.75 | 0.74 - 4.13 | 0.1994 |
